# Eating and Mental Health Disorders

**DOI:** 10.3390/nu18071122

**Published:** 2026-03-31

**Authors:** Hubertus Himmerich

**Affiliations:** 1 Centre for Research in Eating and Weight Disorders (CREW), Department of Psychological Medicine, Institute of Psychiatry, Psychology & Neuroscience, King’s College London, London SE5 8AF, UK; hubertus.himmerich@kcl.ac.uk; 2 South London and Maudsley NHS Foundation Trust, London SE5 8AZ, UK

Recent studies on eating disorders (EDs) and obesity have made substantial progress, leading to an improved understanding of diagnostic entities as well as the psychological, nutritional and biological factors associated with these diseases.

Such innovative scientific and clinical insights have led to the inclusion of avoidant restrictive food intake disorder (ARFID), pica and rumination disorder into the 5th edition of the Diagnostic and Statistical Manual of Mental Disorders (DSM-5) [1], the recognition of the importance of biological factors associated with EDs [2] and awareness of the significance of neurodevelopmental disorders, such as autism spectrum disorder (ASD) and attention deficit hyperactivity disorder (ADHD), for the treatment of EDs [3,4].

However, disturbed eating behavior is not restricted to EDs. A change in food intake can be a symptom of other mental health disorders such as depression [5], or a consequence of psychopharmacological treatment [6]. Obesity is often associated with depression, and a calorie-restricted diet can reduce depressive symptoms in people with obesity [7]. These examples indicate the close association between eating and mental health disorders.

This Special Issue covers psychological, clinical, nutritional, biological and pharmacological aspects of disordered eating, EDs and obesity. The articles build upon advancements in the field and further elucidate the association between eating and mental health disorders.

Psychological and clinical aspects: Four articles of this Special Issue address the topic of depression [8,9,10,11]. Depression is a key mental health problem in people with EDs or obesity, as well as their families. It appears to be closely associated with emotional eating, obesity and food insecurity (FI). In a cross-sectional study, which included more than 700 Peruvian adolescents, Calizaya-Milla et al. found that emotional eating was significantly associated with having depressive symptoms [8]. Bludau et al. reported data on almost 2600 multimorbid patients aged 65 years and older from a prospective multicenter observational cohort study and found a significant prevalence of depression across all body mass index (BMI) classes, with obesity class III showing the highest frequency of cases [9]. Kilpela et al. investigated 295 midlife and older women utilizing a food bank and found that women with FI and binge eating reported significantly worsened anxiety, depression and quality of life compared to women with FI alone [10]. Amianto et al. found that mothers of daughters with anorexia nervosa (AN) exhibited greater harm avoidance and lower self-directedness compared to mothers of daughters without AN, and maternal depressive symptoms were specifically linked to earlier AN onset. These findings suggest that treating maternal depression could be a beneficial preventive strategy [11].

Two articles address eating-related problems associated with neurodiversity [12,13]: In a descriptive qualitative study, Chen et al. conducted focus groups with 12 parents in Hong Kong to explore the eating challenges of their school-age children with ADHD and the parental coping strategies employed. The findings revealed that children’s eating difficulties were linked to ADHD symptoms and educational pressures [12]. Makin et al. provided a review of the literature on autism and ADHD in adults with obesity. They found a potential overrepresentation of ADHD in obese people and ascertained that patients with ADHD and obesity often exhibit increased impulsivity, more psychopathological symptoms and show poorer response to behavioral weight loss programs [13].

Four further articles on psychological aspects report results on identity, personality, body appreciation, cognitive function and FI [14,15,16,17]: A study by Claes et al. investigated the relationship between three identity dimensions and personality disorder (PD) symptoms in 176 female inpatients with an ED. The findings showed that a disturbed identity was strongly associated with cluster B PDs and dependent PD, while a lack of identity predicted cluster A PDs and two cluster C PDs, suggesting that identity is a transdiagnostic feature and that identity-based interventions may benefit patients with co-occurring EDs and PDs [14]. Czepczor-Bernat et al. reported a study in 374 Polish men that investigated whether the combination of high body appreciation and healthy weight was associated with better psychological and motivational profiles compared to low body appreciation and excess weight. They showed that men with high body appreciation and healthy weight reported significantly lower muscle dysmorphia and non-adaptive body attitudes, alongside higher levels of pro-health and adaptive exercise motives [15]. Jowik-Krzemińska et al. compared cognitive function and laboratory parameters in 36 adolescent girls with AN, both during acute malnutrition and after nutritional rehabilitation, against 48 healthy controls. Unexpectedly, in their study, AN patients scored better at both time points than controls in terms of total cognition, attention, estimation, and spatial perception, suggesting the adolescent body maintains a high compensatory capacity for cognitive function despite severe malnutrition [16]. A study by Bryson et al. explored how FI impacted individuals with current or past EDs; the results suggest that FI may exacerbate eating- and food-related symptoms and hinder recovery. Participants reported that FI is associated with low rates of help-seeking due to shame [17].

Nutritional aspects: Three studies in this Special Issue cover the nutritional aspects of eating and mental health disorders [18,19,20]. Çıtar Dazıroğlu et al. reported a cross-sectional study based on data from 907 university students which found that a pro-inflammatory diet, as measured by a higher Dietary Inflammatory Index (DII), was associated with significantly higher depression scores and lower well-being scores. Conversely, a higher Dietary Antioxidant Index (DAI) was linked to significantly lower scores for anxiety, depression, and stress, suggesting that improving the anti-inflammatory potential of the diet may be a beneficial strategy for supporting student mental health [18]. A review paper by Szulc et al. highlighted the significant similarity between orthorexia nervosa (ON) and plant-based diets, noting that while a vegetarian diet may mask ON, rigid dietary adherence can also lead to the misperception of disorder [19]. Dhopatkar et al. wrote a scoping review of 37 studies, examining outcomes associated with Enteral Tube Nutrition (ETN) used for refeeding in AN. They found that ETN usage resulted in similar or higher weight gain compared to oral nutrition, with comparable risks of refeeding syndrome [20].

Biological and pharmacological aspects: Finally, three articles addressed biological and pharmacological aspects of eating- and weight-related disorders [21,22,23]. A study by Žák et al. compared plasma lipids, fatty acids (FAs), and bile acid (BA) profiles in 39 women with restrictive AN and 35 healthy controls. Women with restrictive AN exhibited elevated high density lipoprotein cholesterol (HDL-C), altered FA profiles including lower total polyunsaturated fatty acids (PUFAs) *n*-6 and increased Δ9-desaturase activity, and significantly decreased levels of tauroursodeoxycholic acid (TUDCA), suggesting TUDCA could potentially serve as a biomarker for AN [21]. A systematic review by Micoanski et al. based on 44 case reports on potomania and beer potomania found that these disorders are rare causes of dilutional hyponatremia due to excessive fluid intake, and that patients often present with neurological symptoms like confusion and seizures [22]. A review by Ma et al. summarized the complex pharmacological and clinical effects of natural compounds and mixtures derived from Rosmarinus officinalis, Ginkgo biloba, Bupleurum chinense, and Berberis vulgaris (berberine-containing plants), all traditionally used for mental health and eating-related issues [23]. The information might offer inspiration for the development of novel pharmacological treatments, because approved psychopharmacological options are limited to fluoxetine for bulimia nervosa and lisdexamfetamine for binge eating disorder in some countries [24]. Even though there are case reports about novel ED medications like ketamine [25], randomized controlled trials are lacking.

Table 1 summarizes the psychological traits, psychiatric disorders, social factors, eating behaviors and nutrition-related factors, as well as the treatment strategies mentioned in this Special Issue.

Biomarkers in EDs and obesity: The Special Issue touches on innovations in the field of EDs and obesity. For example, Žák et al. [21] and Micoanski et al. [22] included biological parameters that are associated with disturbed eating and EDs. Regarding biological, and particularly genetic markers, genome-wide association studies (GWASs) across EDs have identified ED-specific but also shared risk genes that suggest a biological vulnerability for EDs [26]. Genetic correlations have implicated both psychiatric and metabolic factors in the origin of EDs [27], and researchers using polygenic risk scores (PRSs) identified a shared genetic background between bipolar I and II disorders, schizophrenia, and AN [28]. Large-scale epigenome-wide association studies have found significant changes in the NR1H3, TNXB, SYNJ2, PRDM16 and HDAC4 genes [29,30,31,32,33]. These genes are relevant for cholesterol metabolism and lipid synthesis (NR1H3), for the structure and stability of connective tissues such as skin, muscles, and joints (TNXB), for membrane trafficking and cell motility (SYNJ2), brown fat differentiation and lipid metabolism (PRDM16) and gene transcription (HDAC4). These findings indicate that AN is closely related to severe mental health disorders like bipolar disorder and schizophrenia but also associated with disorders affecting metabolism and the connective tissue [2].

Structural neuroimaging reveals that AN is associated with white matter, grey matter and global volume loss [34,35,36,37,38,39,40,41,42,43] and changes in subcortical shape, cortical complexity and gyrification [44,45,46,47], whereas BN and BED often show localized changes in the orbitofrontal cortex, an area critical for decision-making and taste preference [48,49,50,51,52,53,54,55,56]. Investigations into functional connectivity indicate that in AN, there is excessive control over hunger, while in BED and BN, the brain’s reward centers often override the cognitive control centers [57,58,59,60,61]. These findings corroborate the relevance of emotional eating and binge eating, as investigated by Calizaya-Milla et al. [8] and Kilpela et al. [10]; their results are included in this Special Issue.

These changes of structure and function in the brain are associated with molecular markers of brain plasticity and brain damage. Levels of leptin [62,63,64,65,66] and BDNF [67] have frequently been found altered in EDs, particularly in AN, suggesting that impaired neuroplasticity may lock patients into repetitive, compulsive behavioral loops. Additionally, fluctuations in Tau protein, neurofilament proteins, and glial fibrillary acidic protein (GFAP) have been studied as potential biomarkers in AN to monitor the neurotoxic effects of malnutrition and inflammatory processes [68,69,70].

Inflammation in eating and mental health disorders: Evidence for the role of inflammatory processes is derived from findings of a bidirectional and shared risk between autoimmune diseases (like type 1 diabetes, celiac disease, Crohn’s disease, and psoriasis) and the development of EDs [71,72,73,74], changes in cytokine concentrations, e.g., tumor necrosis factor (TNF)-α, interleukin (IL)-6, IL-12 and IL-12 in people with EDs [75,76,77,78] and obesity [79,80], changes in white blood cell concentrations [81] and findings of antibody production against molecules and molecule receptors that are involved in appetite regulation and food intake, e.g., α-melanocyte-stimulating hormone (α-MSH) and ghrelin, in people with AN and obesity [82,83,84,85,86,87,88,89,90]. It was even suggested that anti-cytokine medication such as TNF-α and IL-6 blockers could play a future role in the treatment of restrictive EDs such as AN as they have been shown to influence body weight [91,92].

Against this backdrop, it makes sense that Çıtar Dazıroğlu et al. reported in this Special Issue that a pro-inflammatory diet was associated with higher depression scores, whereas an anti-inflammatory diet was linked to lower scores for mental health problems [18]. This finding aligns with previous research that found increased levels of pro-inflammatory cytokines in people with depression [93,94] and in animal models of stress-related [95] and depression-like behavior [96]. Such findings might become relevant in the future as etanercept, a blocker of the pro-inflammatory cytokine TNF-α, has been shown to have anti-depressant effects in animals [96] and to some degree in humans [97]. Chen et al. [12] and Makin et al. [13] linked eating and metabolic health issues to autism and ADHD [13]. Even though the role of inflammation for neurodevelopmental disorders needs to be elucidated further, a recent study suggests an association of neurodevelopmental disorders with prenatal maternal immune activation [98]. Thus, inflammation is likely to play a relevant role in EDs and obesity, depression and neurodevelopmental disorders like ADHD and autism.

Anti-inflammatory natural compounds for the treatment of eating and mental health disorders: Reflecting on the role of inflammation in these disorders, it makes sense that the natural compounds suggested for treatment of eating and mental health disorders by Ma et al. in this Special Issue [23]—Rosmarinus officinalis [99,100,101,102,103,104], Ginkgo biloba [105,106,107,108], Bupleurum chinense [109,110,111] and Berberis vulgaris [112,113,114,115]—have been reported to have anti-inflammatory properties.

Summary: The contributions of this Special Issue suggest that information on depression, maternal depression, autism and ADHD, identity, personality, body appreciation, cognition, FI and a person’s diet and drinking habits should inform the individual psychiatric formulation for the treatment of a person with an eating or mental health disorder. Biomarkers like body weight, HDL-C, Δ9-desaturase, PUFA n-6 and TUDCA might help to assess nutritional health. In severe cases of restrictive EDs, ETN might be a time-limited clinical option. Rosmarinus officinalis, Ginkgo biloba, Bupleurum chinense, and Berberis vulgaris have been suggested as traditional remedies for the treatment of eating and mental health disorders.

## Figures and Tables

**Table 1:** Psychological and nutritional risk factors as well as therapeutic strategies mentioned in this Special Issue.

Psychosocial Risk Factors	Eating and Nutrition	Treatment Strategies
Psychological traits - Increased harm avoidance - Low self-directedness - Disturbed/lack of identity - Low body appreciation Psychiatric disorders - Personality disorder - Depression - ADHD - Orthorexia nervosa Social factors - Food insecurity - Educational pressure - Low quality of life	Eating behaviors - Emotional eating - Binge eating - Excessive fluid intake - Pro-inflammatory diet Nutritional risk factors - Extreme body weight - HDL-C ↑ - Δ9-desaturase ↑ - PUFA n-6 ↓ - TUDCA ↓	Treatment of comorbidities - Depression - ADHD Nutritional strategies - Oral refeeding - Enteral Tube Nutrition Natural compounds - Rosmarinus officinalis - Ginkgo biloba - Bupleurum chinense - Berberis vulgaris

Abbreviations: Attention deficit hyperactivity disorder (ADHD), high-density lipoprotein cholesterol (HDL-C), polyunsaturated fatty acid (PUFA), tauroursodeoxycholic acid (TUDCA).
